# Commentary: Distinct neural mechanisms for remembering when an event occurred

**DOI:** 10.3389/fpsyg.2017.00189

**Published:** 2017-02-21

**Authors:** Sarah DuBrow, Lila Davachi

**Affiliations:** ^1^Department of Psychology, New York UniversityNew York, NY, USA; ^2^Center for Neural Science, New York UniversityNew York, NY, USA

**Keywords:** temporal order memory, temporal context, sequence memory, recency discrimination, hippocampus

Memory for the relative order of events is a critical feature of episodic remembering that is thought to rely on hippocampal processes (Eichenbaum, 2013; Davachi and DuBrow, 2015). However, there are multiple ways in which the hippocampus may support order memory. Here, we review a recent fMRI paper by Jenkins and Ranganath (2016) investigating two potential memory mechanisms that may support recency discrimination. To briefly summarize, participants were scanned while encoding sequences of object images and were subsequently tested on which of two objects had been presented more recently. The authors examined neural patterns during encoding that predicted later recency judgments and found evidence that item strength and context differentiation support order memory. Our goal here is to provide a theoretical perspective on these and related findings to highlight how numerous mechanisms may support order memory and how fMRI can be leveraged to test competing theories.

Perhaps the most intuitive way to evaluate the order of two items is to compare how strong they are in memory. Since memory strength decays over time, an item's current strength can provide an estimate of how much time passed since it was encountered (Hinrichs, 1970). To determine which of two items occurred more recently, one strategy might be to simply select the one that has the higher activation strength (Hintzman, 2005, c.f. Hintzman, 2010). Jenkins and Ranganath (2016) found evidence in line with a strength-based temporal representation in the prefrontal (PFC) and medial temporal lobe cortices including the perirhinal cortex, which has been consistently implicated in encoding item strength (Aggleton and Brown, 1999; Davachi, 2006; Diana et al., 2007; Eichenbaum et al., 2007). Specifically, these regions showed greater activation during the initial encoding of items later endorsed as more recent regardless of their true temporal position. While these results are consistent an item-strength comparison account of recency judgments, an alternative retrieval process called scanning could show similar effects at encoding. Backwards scanning models propose that memoranda are sequentially sampled from the end until reaching an item with a sufficient match to one of the recency probes (Hacker, 1980; Howard et al., 2015). Thus, if the more recent item was not encoded strongly enough, it could be bypassed in favor of the stronger, earlier item, consistent with the findings of Jenkins and Ranganath.

Another possibility is that recency judgments could be supported by a comparison of the contexts associated with the objects during encoding. Prominent memory theories propose that items are bound to a temporal context representation that gradually changes over time (Howard and Kahana, 2002; Polyn et al., 2009). Jenkins and Ranganath suggest that this representation may be used to guide recency judgments, presumably by a process that compares the retrieved contexts of the two items and selects the item whose associated context is most similar to the current state. The more differentiated the two retrieved contexts are, the easier it should be to make the comparison. The authors find evidence for this “context differentiation” account in bilateral hippocampus as well as in regions of the medial and anterior PFC. Specifically, the more dissimilar the fMRI patterns were during the encoding of the two items, the better performance was on later recency discrimination. Assuming that pattern dissimilarity reflects a change in the intervening context above and beyond differences between the items themselves, this suggests that context differentiation leads to better order memory because the items' contexts are more discriminable at retrieval. Note, however, that neural patterns at retrieval were not examined.

The item strength and context differentiation accounts of order memory are similar in that they are both based on estimating and comparing distances of the two items between encoding and retrieval. However, there are at least two other major classes of theories of temporal representation—those that are based on the absolute time or position at which an event occurred (i.e., location-based) and those that are based on relative time or position (Friedman, 1993). One example of a model that encodes relative position is associative chaining, in which each item in a sequence is directly linked to its neighbors (e.g., Lewandowsky and Murdock, 1989). The temporal context theories described above are actually closely related to associative chaining. However, rather than employing direct item-item links, temporal context theory proposes that neighboring items are linked indirectly through their associated context representation (Howard and Kahana, 2002). These associations allow an item's retrieved context to elicit retrieval of nearby items that share a similar temporal context. Thus, an alternate account of temporal context in recency judgments might predict that retrieving the context associated with the recency items may lead to the reactivation of the intervening sequence, since the intervening items share context with both recency probes. These sequential associations may in turn provide the relative order information necessary to make accurate recency judgments. Note, a similar retrieval process applied to a location-based temporal representation (e.g., Howard et al., 2015) could also retrieve sequential associations with more absolute temporal precision.

There is evidence supporting this associative account of recency discrimination in episodic memory from both behavioral and fMRI work. Behaviorally, intervening boundaries have been shown to disrupt associative binding (Zwaan and Radvansky, 1998; Ezzyat and Davachi, 2011) and impair order memory (DuBrow and Davachi, 2013; Horner et al., 2016). There is also evidence that, when making recency judgments, the intervening sequence is incidentally reactivated (DuBrow and Davachi, 2013, 2014). Importantly, in this design, hippocampal pattern *similarity* was related to successful recency judgments (DuBrow and Davachi, 2014) in contrast to the hippocampal pattern *dissimilarity* reported by Jenkins and Ranganath. One possibility is that these conflicting results may be due to differences in the processes engaged during encoding. DuBrow and Davachi promoted the use of associative encoding, which has been shown to influence behavioral and neural order memory effects (Konishi et al., 2006; Jonker and Macleod, 2016). In contrast, the use of a single stimulus category in Jenkins and Ranganath may have promoted a differentiation strategy. Indeed, hippocampal differentiation of items that share similar features or associates has been shown to lead to better memory (LaRocque et al., 2013; Hulbert and Norman, 2015; Schlichting et al., 2015; Favila et al., 2016). Thus, it is not clear to what extent hippocampal patterns in these studies indexed context *per se*, as opposed to processes that either promote maintenance (pattern similarity) or differentiation (pattern dissimilarity).

The study by Jenkins and Ranganath is an important contribution to the literature on temporal memory (for recent reviews, see Howard and Eichenbaum, 2013; Eichenbaum, 2014; Davachi and DuBrow, 2015; Ranganath and Hsieh, 2016). Together with previous data, this work suggests that no singular mechanism supports all order memory, but instead multiple temporal representations and retrieval mechanisms may coexist. This work also highlights the importance of considering how distinct cognitive processes that can be localized to the same brain region may give rise to similar behaviors, in this case successful order memory, despite different underlying mechanisms. Indeed, while a wealth of data implicates the hippocampus in temporal memory, the mechanisms attributed to it have been wide ranging and include each class of theories discussed above—relative distance supported by context differentiation (Manns et al., 2007; Ezzyat and Davachi, 2014; Jenkins and Ranganath, 2016), relative order supported by sequential binding (Tubridy and Davachi, 2011; Schapiro et al., 2012; DuBrow and Davachi, 2014), and location information supported by positional coding (Hsieh et al., 2014; Kalm and Norris, 2014). In the Jenkins and Ranganath study alone, the hippocampus showed both item strength and context differentiation effects at encoding. Another recent study showed that associative and distance-based order judgments engaged the hippocampus equally (Lieberman et al., 2016). Moving forward by using explicit models to compare the predictions of different temporal memory theories will help specify the precise computational role(s) of a given brain region (e.g., Kalm and Norris, 2014). In addition, collecting data during both encoding and retrieval would allow the underlying *representation* of temporal order to be evaluated separately from the decision *process*, and in turn, capture more individual variability in order memory judgments. Manipulating access to temporal information within the same study will also be necessary to determine whether different mechanisms could be employed adaptively depending on available sources of information and current retrieval goals. For example, lengthening the interval between items may flip the relative reliance on associative vs. distance-based information and may be indexed by the influence of pattern similarity vs. dissimilarity, respectively, on accuracy. Ultimately, examining whether and how these processes may tradeoff at different timescales and under various encoding and retrieval conditions will be critical for establishing a comprehensive model of temporal memory.

## Author contributions

All authors listed, have made substantial, direct and intellectual contribution to the work, and approved it for publication.

## Funding

This work is supported by the National Institute of Mental Health (RO1-MH074692) and the National Science Foundation (DGE 0813964).

### Conflict of interest statement

The authors declare that the research was conducted in the absence of any commercial or financial relationships that could be construed as a potential conflict of interest.
